# *N*-(2-Hydroxy)-propyl-3-trimethylammonium, *O*-Mysristoyl Chitosan Enhances the Solubility and Intestinal Permeability of Anticancer Curcumin

**DOI:** 10.3390/pharmaceutics10040245

**Published:** 2018-11-20

**Authors:** Daniella S. Silva, Danilo M. dos Santos, Andreia Almeida, Leonardo Marchiori, Sérgio P. Campana-Filho, Sidney J. L. Ribeiro, Bruno Sarmento

**Affiliations:** 1 Institute of Chemistry, São Paulo State University—UNESP, Araraquara 4800-900, Brazil; danyss8@hotmail.com (D.S.S.); leonardo.marchiori.lm@gmail.com (L.M.); sidney.jl.ribeiro@unesp.br (S.J.L.R.); 2 Embrapa Instrumentação, Rua XV de Novembro 1452, São Carlos 13560-970, Brazil; danilomartins_1@hotmail.com; 3 Institute for Research and Innovation in Health (i3S), Rua Alfredo Allen, 208, 4200-393 Porto, Portugal; andreia.almeida@ineb.up.pt; 4 ICBAS—Institute of Biomedical Sciences Abel Salazar, University of Porto, Rua de Jorge Viterbo Ferreira, 228, 4050-313 Porto, Portugal; 5 CESPU—Institute for Research and Advanced Training in Health Sciences and Technologies, Rua Central de Gandra, 1317, 4585-116 Gandra, Portugal; 6 Sao Carlos Institute of Chemistry, University of Sao Paulo—USP, Av. Trabalhador São-Carlense, 400, São Carlos 13566-590, Brazil; scampana@iqsc.usp.br

**Keywords:** chitosan derivatives, amphiphilic polymers, polymeric micelles, quaternization, curcumin, intestinal delivery

## Abstract

An amphiphilic derivative of chitosan containing quaternary ammonium and myristoyl groups, herein named as ammonium myristoyl chitosan (DMCat), was synthesized by reacting glycidyltrimethylammonium chloride (GTMAC) and myristoyl chitosan (DMCh). The success of the modification was confirmed using Fourier-transform infrared spectroscopy (FTIR) and ^1^H nuclear magnetic resonance (NMR) spectroscopy. The average degrees of alkylation and quaternization ($\bar{DQ}$) were determined by using ^1^H NMR and conductometric titration. The zeta potential of the micelles was higher than 28 mV while its average size and encapsulation efficiency ranged from 280 nm to 375 nm and 68% to 100%, respectively. The in vitro cytotoxicity of the unloaded and curcumin (CUR)-loaded micelles was tested against Caco-2 and HT29-MTX intestinal epithelial cell lines. The results showed no cytotoxic effect from loaded and unloaded micelles as compared to free CUR. In the permeability test, it was observed that both types of micelles, i.e., DMCh and DMCat, improved CUR permeability. Additionally, higher permeability was verified for both systems in Caco-2/HT29-MTX:Raji B because of the mucoadhesive character of chitosan and its ability to open tight junctions. The results indicated that DMCat micelles, due to the physico-chemical, improved characteristics may be a promising carrier to encapsulate CUR aiming cancer therapy.

## 1. Introduction

Polymeric micelles have attracted considerable interest for controlled drug delivery over the last few years, due to their unique properties and behaviors resulting from their small size and high load capacity [1]. These nanostructured materials show potential to deliver chemotherapeutics to tumor cells maintaining their concentration at the desired site for a sufficient time to have a therapeutic effect, increasing the efficacy of treatment and reducing the toxicity [2].

Curcumin (CUR) is a naturally occurring phytochemical that has been widely used as a therapeutic agent against a variety of cancers [3,4,5,6,7,8]. In particular, CUR is a cheap compound (especially when compared with popular anticancer agents like paclitaxel and camptothecin) and has properties in antioxidant, anti-inflammatory, antimicrobial, anticancer, anticarcinogenic, chemopreventive, chemotherapeutic, and chemosensitizing activities [9,10]. Although CUR possesses remarkable medicinal properties, its bioavailability during cancer treatments is limited due to its low solubility and poor stability in aqueous solution. Other than its rapid degradation in physiological conditions, however, there is a wide interest in the development of systems to solve problems related to the limitations of the applicability of CUR as a therapeutic agent [4,5,6,7,11]. To enhance solubility, stability, pharmacokinetics profile of curcumin and thus its therapeutic efficiency, various drug delivery systems have been investigated, including nanoemulsions, nanosuspensions, and polymeric micelles [11,12].

In recent years, drug delivery systems based on polymeric micelles have been the subject of research. Several formulations are undergoing clinical trials due to the ability of the micelles to encapsulate hydrophobic and hydrophilic drugs [13,14,15]. In addition, these systems have shown greater physical stability and encapsulation efficiency, when compared to liposomes [16,17,18,19,20]. Shell/core polymeric micelles, in particular, exhibit advantages regarding encapsulation and controlled release of drugs since their hydrophobic nuclei promote the solubilization of hydrophobic actives, avoiding drug degradation. On the other hand, hydrophilic outer shells avoid recognition by the immune system, prolonging the circulation time of the drug in the body [21,22].

Lately, micelles formulations based on chitosan have presented promising results in the drug delivery field [23,24,25,26,27,28]. This cationic biopolymer exhibits biological activities, such as biocompatibility, biodegradability, mucoadhesiveness, and antimicrobial activity, in addition to presenting antioxidant properties [29,30,31]. Chitosan has present in its backbone reactive groups (–OH and –NH_2_) that make it susceptible to chemical modifications. Various derivatives of chitosan are obtained via the addition of hydrophobic groups with different degrees of substitution (DS). In particular, chitosan modified with stearic acid (DS = 4.96%) [32] and linoleic acid (DS = 1.8%) [33] exhibit self-assembly and loading abilities, which improve the solubility of hydrophobic drugs [34,35]. However, the addition of a small portion of hydrophobic units makes chitosan extremely insoluble in neutral medium, leading to a low loading capacity of the micelles for non-ionic hydrophobic drugs [34,36].

Previous studies with the structural modification of chitosan via an *O*-acylation reaction with myristoyl chloride showed excellent capacity for encapsulation and solubilization of hydrophobic drugs, such as paclitaxel [37]. This is due to the affinity of the acyl chains within the drug. However, because such a derivative exhibited a low degree of substitution (DS = 6.8%), the characteristics of the starting chitosan have been maintained, and its limited solubility at a neutral pH has hindered its application. Thus, the limitations resulting from the modification of chitosan with myristoyl chloride via an *O*-substitution reaction can be solved by carrying out a further chemical modification.

The introduction of hydrophilic and hydrophobic groups has shown excellent results in the encapsulation of hydrophobic drugs, such as paclitaxel and doxorubicin. In this sense, different amphiphilic derivatives of chitosan have been synthesized, such as *N*-octyl-*N*, *O*-carboxymethylchitosan [18], colic acid grafted *N*-(2,3-(dihydroxypropyl) chitosan [38], chitosan-*g*-polylactic [39], *N*-octyl-*N*-trimethylchitosan [40], *N*-propyl-*N*-methylenephosphonic chitosan [41], *N*-naphthyl-*N*, *O*-succinylchitosan [42]. In all of these studies, amphiphilic derivatives of chitosan are suggested to be promising efficient carriers for hydrophobic drugs, especially when compared to hydrophobically modified chitosan.

**Scheme 1:**
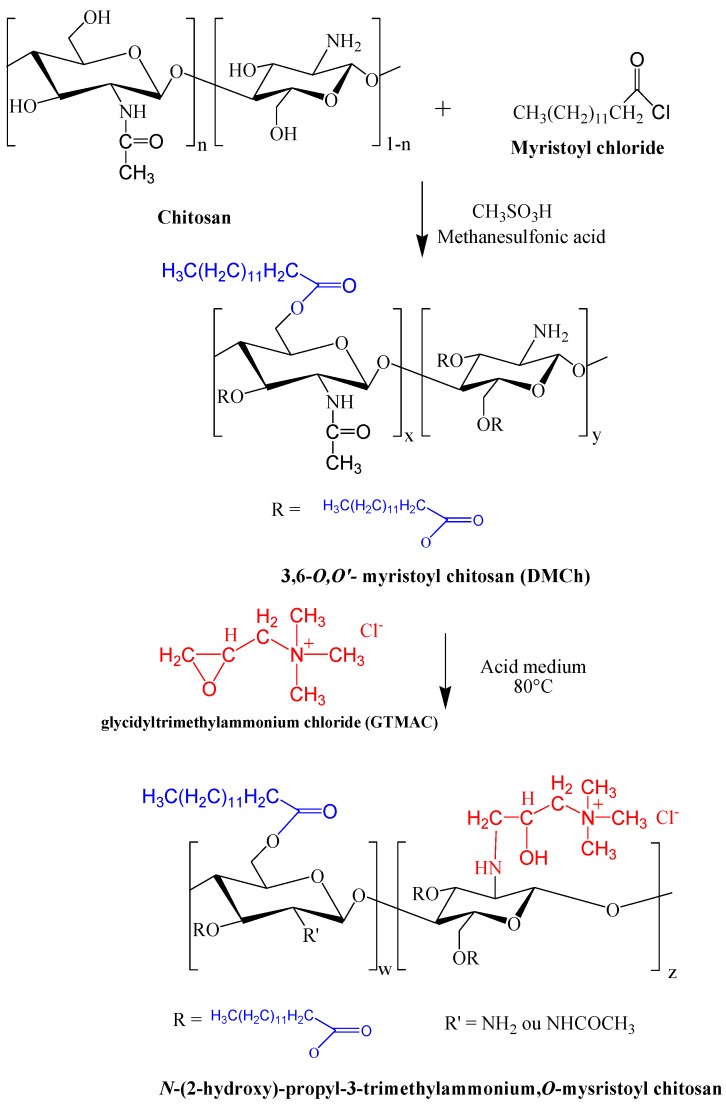
The synthesis of *N*-(2-hydroxy)-propyl-3-trimethylammonium,*O*-mysristoyl chitosan.

This work aims to develop a new amphiphilic derivative of chitosan via *O*-acylation and quaternization reactions in chitosan (Scheme 1), since the first step (*O*-acylation) has already been conducted with excellent results for anticancer drug delivery [37]. Studies showed that the synthesis of quaternized chitosan via *N*-substitution by reacting it with *N*-(2-hydroxyl) propyl-3-trimethyl ammonium chloride presented properties of interest, such as water solubility, antibacterial activity, mucoadhesiveness, increased permeability to hydration, and strong stereochemical hindrance of quaternary amine groups [43,44,45]. 

In this study, an amphiphilic derivative of chitosan containing quaternary ammonium and myristoyl goups was prepared and its ability to form self-assembled micelles with ability to encapsulate curcumin was explored. The efficiency of the structural modification was evaluated by FTIR and ^1^H nuclear magnetic resonance (NMR) spectroscopy and conductometric titration. The physical-chemical characteristics such as average size, zeta potential, drug loading efficiency, and critical aggregation concentration (CAC) were determined. The in vitro cytotoxicity was evaluated using Caco-2 (clone C2BBe1) and HT29-MTX cell lines, as well as the in vitro intestinal permeability.

## 2. Materials and Methods

### 2.1. Materials

Chitosan acquired from a commercial supplier (Cheng Yue Plating Co. Ltd.; Pequim, China) was purified according to Santos et al. [46], and then dried at 30 °C. The average degree of acetylation ($\bar{DA}$) of chitosan was 5%, as determined by the ^1^H NMR analysis. The viscosity average molecular weight ($\bar{Mv}$) of chitosan was calculated from its intrinsic viscosity, which was determined in 0.3 mol L^−1^ acetic acid/0.2 mol L^−1^ sodium acetate buffer (pH 4.5) at 25.00 ± 0.01 °C, by using the Mark-Houwink-Sakurada equation, resulting in $\bar{Mv}$ ≈ 87.000 g mol^−1^ [46,47]. Glycidyltrimethylammonium chloride (GTMAC) was acquired from Sigma-Aldrich (Saint Louis, MO, USA). All other reagents and solvents were used as acquired, i.e., without any purification.

### 2.2. Synthesis of 3,6-O,O’-Myristoyl Chitosan

The synthesis of 3,6-*O*,*O*′-myristoyl chitosan (DMCh) was realized by adding 1 g of chitosan in 25 mL of methanesulfonic acid (MeSO_3_H). In addition, an amount of myristoyl chloride was added in order to generate an excess of chitosan (≈13.3). After 1 h of reaction, the product was (i) neutralized with 5% (*w*/*v*) aqueous sodium bicarbonate solution, (ii) centrifugated, (iii) extensively washed with distilled water, (iv) purified in Soxhlet, and finally, (v) dried in a vacuum oven at room temperature. This procedure can be found in detail in a previous work [37].

### 2.3. Synthesis of N-(2-Hydroxy)-Propyl-3-Trimethylammonium Chitosan Chloride

The reaction of glycidyltrimethylammonium chloride (GTMAC) and DMCh in an acid medium was carried out as described in the literature [46] to result in *N*-(2-hydroxy)-propyl-3-trimethylammonium,*O*-mysristoyl chitosan chloride. Briefly, 1 g of DMCh was dissolved in 60 mL of acetic acid 1% (*v*/*v*), until complete dissolution. An aqueous solution of GTMAC was added dropwise to the DMCh solubilized; the reaction mixture was then stirred in a glycerin bath for 8 h, under constant stirring at 80 °C. Following, excess acetone was added to the reaction medium to result in the precipitation of the product, which was filtered. Finally, the solid was purified by extraction with acetone in Soxhlet during 6 h and dried in a vacuum oven (30 °C), obtaining as final product the DMCat polymer.

### 2.4. Preparation of CUR-Loaded DMCh and DMCat Micelles

Both CUR-loaded DMCh and DMCat micelles were prepared by following the steps described in earlier works [37,48,49]. In essence, DMCh is dissolved in acetic acid 0.1% while DMCat is dissolved in deionized water. CUR was dissolved in acetone (1 mg/mL). Next, aliquots containing 50, 100 and 150 μg of CUR were added in the different polymer solutions (DMCh and DMCat). Then, the solutions are magnetically stirred at 300 rpm for 2 h (room temperature) and freeze-dried. Finally, the CUR-loaded micelles are resuspended and separated by centrifugation at 5000 rpm for 10 min.

### 2.5. Characterization

#### 2.5.1. Fourier-Transform Infrared Spectroscopy (FTIR)

FTIR was performed on an IRAffinity Shimadzu spectrometer (Shimadzu Scientific Instruments, Columbia, MD, USA) in a 4000–400 cm^−1^ wavelength range, with an accumulation of 32 scans and resolution of 4 cm^−1^. In order to prepare the pellets, the sample and KBr were dried at 60 °C for 8 h and mixed in a ratio of 1:100 (sample:KBr).

#### 2.5.2. ^1^H NMR Spectroscopy

A Bruker AVANCE III spectrophotometer 400 MHz (Bruker, Billerica, MA, EUA) was used to obtain ^1^H NMR spectra, aiming the samples’ characterization. Basically, the Equations (1)–(3) are used to determine the average degree of acetylation of chitosan ($\bar{DA}$) ([50,51]), degree of substitution ($\bar{DS}$) of the derivative 3,6-*O*,*O’*-myristoyl chitosan ([37]) and degree of quaternization ($\bar{DQ}$) of the derivative *N*-(2-hydroxy)-propyl-3-trimethylammonium, *O*-mysristoyl chitosan ([46]), respectively. In other words, $\bar{DA}$, $\bar{DS}$, and $\bar{DQ}$ refers to the amount of acetylated units present in chitosan, as well as both the myristoyl groups added to chitosan and the quaternary groups present in derivative *N*-(2-hydroxy)-propyl-3-trimethylammonium,*O*-mysristoyl chitosan.

(1) DA¯(%)=(ACH33AH2−H66)∗100 

*A_CH_*_3_ = area of the methyl hydrogens of the acetamido group (of GlcNAc units);

*A_H_*_2–*H*6_ = area of hydrogens bonded to the C (2–6) carbon of the glycopyranose ring.

(2) DS¯(%)=(AMe3AH2−H66)∗100 

*A_Me_* = area of the methyl hydrogens of the myristoyl group;

*A_H_*_2–*H*6_ = area of hydrogens bonded to the C (2–6) carbon of the glycopyranose ring.

(3) $$\bar{DQ}\left(\%\right)\;=\frac{IH1\prime }{IH1^{\prime }+IH1}\;\cdot 100\;$$

*I_H_*_1′_= the integral of the signal due to the anomeric hydrogen bonded to *N*-substituted GlcN (units);

*I_H_*_1_ = is the integral of the signal due to the anomeric units hydrogen bonded to unsubstituted GlcN (units).

#### 2.5.3. Conductometric Titration

The $\bar{DQ}$ of DMcat samples were also determined by dosing the counter-ions Cl^−^ through titration with standardized 0.017 mol L^−1^ aqueous AgNO_3_ solution. Thus, the quaternized derivative (0.1 g) was dissolved in deionized water (100 mL) and the conductivity of the solution was measured at 25.00 ± 0.01 °C as a function of the added volume of aqueous AgNO_3_ by using a Handylab LF1 conductivimeter (SI Analytics, Mainz, Germany). The value of $\bar{DQ}$ of samples was calculated from the titration curves according to [46].

#### 2.5.4. Critical Aggregation Concentration (CAC)

The conductivity method was performed to determine the CAC by using a Consort C863 conductivity meter. In order to prepare the samples, different polymer concentrations were utilized (1 × 10^−6^ mg/mL ≤ *C*p ≤ 1 mg/mL). The DMCh sample was solubilized in acid medium due to its limited solubility in neutral medium, while deionized water was used to dissolve DMCat.

#### 2.5.5. Particle Size and Surface Charge

The average size, polydispersity, and zeta potential of DMCh (in acid medium) and DMCat (in water) micelles were determined using dynamic light scattering (DLS) measurements (ZetaSizer Nano ZS, Malvern, UK). Three replicates of each formulation were produced and analyzed. 

#### 2.5.6. Drug Encapsulation Efficiency

The drug encapsulation efficiency (EE%) was quantified by measuring the absorbance of the CUR solution at 340 nm using a UV-Vis spectrophotometer [3,7]. The measurements were performed by an indirect method in triplicate, in which the unencapsulated drug was solubilized in acetone and quantified. The amount of CUR loaded into the DMCh and DMCat micelles was determined according to Equation (4):

(4) $$\;\mathrm{EE}\%=\frac{\left({\mathrm{CUR}}_{\mathrm{total}}-{\mathrm{CUR}}_{\mathrm{free}}\right)}{{\mathrm{CUR}}_{\mathrm{total}}}\times 100\;$$

### 2.6. In Vitro Studies

#### 2.6.1. Cell Culturing Reagents

Caco-2 cells (clone C2BBe1) were obtained from American Type Culture Collection (ATCC, Manassas, VA, USA) and HT29-MTX cell line was kindly provided by Dr. T. Lesuffleur (INSERMU178, Villejuif, France). Dulbecco’s Modified Eagle Medium (DMEM, Lonza, Basel, Switzerland), supplemented with 10% (*v*/*v*) fetal bovine serum (FBS, Merck Millipore, USA), 1% (*v*/*v*) penicillin (100 UI/mL, Merck Millipore), streptomycin (100 μg/mL, Merck Millipore), and 1% (*v*/*v*) nonessential amino acids (NEAA, Merck Millipore). 3-(4,5-dimethylthiazol-2-yl)-2,5-diphenyltetrazolium bromide (MTT), dimethyl sulfoxide (DMSO) and Triton X-100 were obtained from Sigma Aldrich.

#### 2.6.2. Cytotoxicity Assay

The cytotoxicity of CUR-loaded micelles and free CUR was assessed in Caco-2 and HT29-MTX cell lines, using the MTT reagent. Cells were seeded (200 μL) into 96-well plates at a density of 1 × 10^4^ cells/well for HT29-MTX cell line and 2 × 10^4^ cells/well for Caco-2 and then incubated during 24 h in a Binder^®^ incubator at 37 °C with a humidified atmosphere and 5% CO_2_ to reach exponential growth prior to the assay test. On the following day, the medium was removed and the cells were washed twice with 200 μL of phosphate buffered saline (PBS). Next, the cells were treated with free CUR and CUR-loaded and unloaded micelles at the concentrations of 5, 10, 20, and 50 μg/mL. The negative control, which were cells treated with cell culture medium and positive control and treated with 1% (*w*/*v*) Triton X-100 in DMEM [52]. The cultured cells were incubated for 4 h in the presence of different concentrations of the referred samples. After this period, the wells were washed twice with PBS at 37 °C and 200 μL of the MTT reagent (0.5 mg/mL in cell culture medium) was added to each well, followed by an incubation period of 4 h. After, the MTT was removed and 200 μL of DMSO was added to each well to dissolve the formazan crystals. The plates were shaken for 10 min inside the microplate reader before the relative color intensity has been measured at 570 nm and taking the absorbance at 630 nm as a reference, by using a microplate reader (Synergy 2, Biotek Instruments Ltd., Winooski, VT, USA). The percentage of cell viability was calculated according to the Equation (5):

(5) $$\;\mathrm{Cell}\;\mathrm{viability}\;\left(\%\right)=\frac{\mathrm{experimental}\;\mathrm{value}-\mathrm{negative}\;\mathrm{control}}{\mathrm{positive}\;\mathrm{control}-\mathrm{negative}\;\mathrm{control}}\times 100\;$$

#### 2.6.3. Permeability Studies

Monoculture model of Caco-2 cells and triple co-culture model of Caco-2/HT29-MTX:Raji B cells were seeded on 6-well Transwell^TM^ cell culture inserts (transparent PET, 3 μm pore size, 4.67 cm^2^, Corning Life Sciences, New York, NY, USA), according to Araújo. F [53,54]. The cell lines were seeded on the apical compartment at a density of 1 × 10^5^ cells/cm^2^. In the case of the triple co-culture model, Caco-2 and HT29-MTX were seeded at the proportion 90:10 and at the 14th day of culture, Raji B cells were added to the basolateral compartment in a density of 1 × 10^5^ cells/cm^2^. Cells were grown for 21 days until reaching confluency and the medium was changed every 2 days.

Before permeability experiments, cells were washed twice with pre-warmed Hank’s buffered salt solution (HBSS) and then replaced with new HBSS and allowed to equilibrate for 30 min at 37 °C at 100 rpm. Permeability studies of CUR-loaded micelles and free CUR (20 μg/mL) were run at similar conditions during 3 h and samples were collected at different time (15, 30, 60, 90, 120, and 180 min). Each sample was taken from the basolateral side of the Transwell^TM^ cell culture insert, which contained 1% (*v*/*v*) DMSO in HBSS in order to maintain the sink conditions. The same volume of pre-warmed 1% (*v*/*v*) DMSO in HBSS was added to replace the withdrawn volume. The cell monolayers integrity was measured at the during 21 days of growth and during the experiment, using an epithelial voltohmmeter EVOM^2®^ with chopstick electrodes (World Precision Instruments, Sarasota, FL, USA). The transepithelial electrical resistance (TEER) values, expressed in percentage, were normalized in function of the TEER value after equilibrium. All experiments were performed in triplicate and the CUR was quantified by measuring the absorbance at 340 nm as referred previously.

### 2.7. Statistical Analysis

The experiments were performed in triplicate and are represented as mean ± standard deviation (SD). A two-way ANOVA with Bonferroni multiple/post hoc group comparisons was used to analyze the cytotoxicity and permeability data. GraphPadPrism software (GraphPad Software, San Diego, CA, USA) was used and the level of significance was set at probabilities of * *p* < 0.05, ** *p* < 0.01, *** *p* < 0.001 and **** *p* < 0.0001.

## 3. Results and Discussion

### 3.1. Spectroscopic Characterizatio 

**Figure 1:**
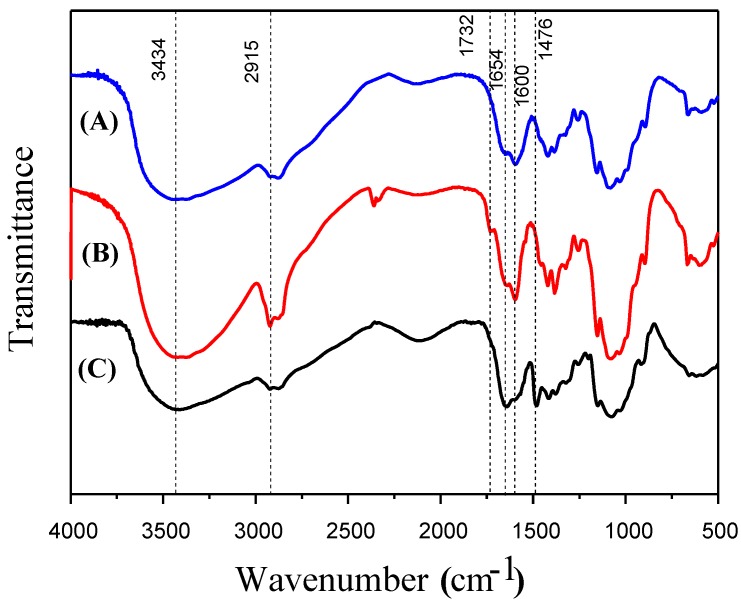
(**A**) Infrared spectra of chitosan, (**B**) DMCh, and (**C**) DMCat.

The infrared spectra of chitosan, DMCh, and DMCat derivatives (Figure 1) were characterized by an intense and broad band centered at 3434 cm^−1^, associated to the axial stretching of hydroxyl and amine groups; a band at 2915 cm^−1^ corresponding to stretching vibration of CH; the bands at 1654 and 1600 cm^−1^ assigned to the C=O stretching and N–H bending, respectively [55]. The spectrum of DMCh (Figure 1B) also exhibits a weak band centered at 1732 cm^−1^, due to axial deformation of carbonyl ester that is related to the *O*-acylation of chitosan [37]. In the spectrum of DMCat, a new band is observed at 1483 cm^−1^, which is attributed to the CH bending of ^+^N(CH_3_)_3_ group [56]. Additionally, the band corresponding to the primary amine observed at 1600 cm^−1^ in the spectrum of chitosan and DMCh is less intense in the spectrum of DMCat and it is shifted to lower wavenumber while that band observed at 1654 cm^−1^ is more intense in the spectra of DMCat, which confirms that the primary amine group of GlcN units of DMCh has been modified to secondary amine group as a consequence of the reaction with GTMAC [46,57]. 

**Figure 2:**
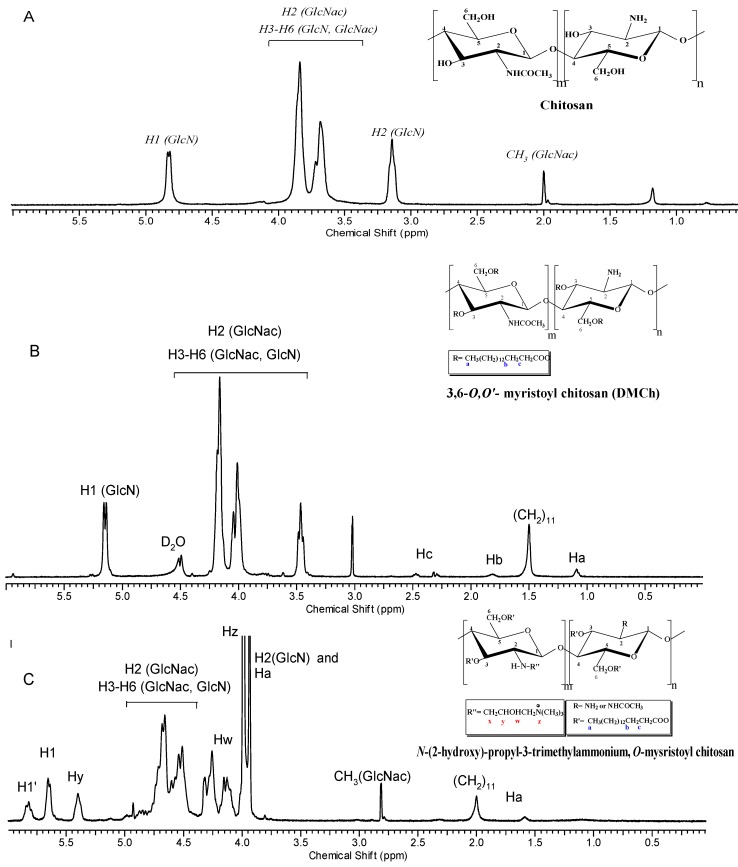
(**A**) ^1^H nuclear magnetic resonance (NMR) spectra of chitosan, (**B**) DMCh, and (**C**) DMCat in solution D_2_O/HCl 1% (*v*/*v*) acquired at 80 °C.

The comparison of the ^1^H NMR of chitosan (Figure 2A), DMCh (Figure 2B), and DMCat (Figure 2C) provide additional evidences for the structural modifications resulting of *O*-acylation of chitosan and the quaternization of DMCh. The ^1^H NMR spectrum of chitosan exhibited singlets at 2.0 ppm and 3.2 ppm characteristics of methyl hydrogens of GlcNAc units and to the hydrogen bonded to C2 of GlcN units, respectively. The set of signals in the range of 3.3–4.0 ppm is attributed to the hydrogens H3–H6 from GlcN unit and the hydrogen bonded to C2 of GlcNAc unit, while that signal occurring at 4.80 ppm is due to the hydrogen (H1) bonded to the anomeric carbon (C1) [46]. The average degree of deacetylation of chitosan was calculated from its ^1^H NMR spectrum by taking into account the intensity of the signals due to methyl hydrogens from acetamido groups (2.0 ppm) and due to H2–H6 of GlcN/GlcNAc units (3.3–4.0 ppm) and resulted in $\bar{DA}$ = 5%. 

The ^1^H NMR spectrum of DMCh (Figure 2B) exhibits additional signals at 1.1, 1.5, 1.8, 2.5, and 3.1 ppm corresponding to the hydrogen atoms of CH3, CH2, CH2 (β), and CH2 (α) moieties of myristoyl group. The peaks at 3.2–4.2 ppm refer to the hydrogens H3–H6 from the GlcN unit and the hydrogen bonded to C2 of the GlcNAc unit [37]. The $\bar{DS}$ of DMCh was calculated from ^1^H NMR spectrum, as described in the Section 2.5.2, and resulted in $\bar{DS}$ = 6%. As a consequence of quaternization of DMCh, the ^1^H NMR spectrum of DMCat (Figure 2B) showed signals at 4.0 ppm and 4.2 ppm assigned to the methyl hydrogens of N^+^(CH_3_)_3_ and methylene hydrogens of NHCH_2_, respectively, which were due to the introduction of the substituent on the chitosan chains. Additionally, the signals of the hydrogens H3–H6 from GlcN unit and the hydrogen bonded to C2 of GlcNAc unit shifted from 3.2–4.2 ppm to 4.4–4.9 ppm upon the chemical modification of DMCh. The signals at 5.6 ppm and 5.7 ppm are ascribed to the hydrogen bonded to the anomeric carbon of unsubstituted and substituted GlcN units, respectively. The $\bar{DQ}$ was calculated from the corresponding ^1^H NMR spectrum by using Equation (2) and resulted in $\bar{DQ}$ = 34.7%. The average degree of quaternization of DMcat was also determined from conductometric titration and resulted in $\bar{DQ}$ = 36%, thus demonstrating a good agreement with that calculated from the ^1^H NMR spectra.

### 3.2. Critical Aggregation Concentration

**Figure 3:**
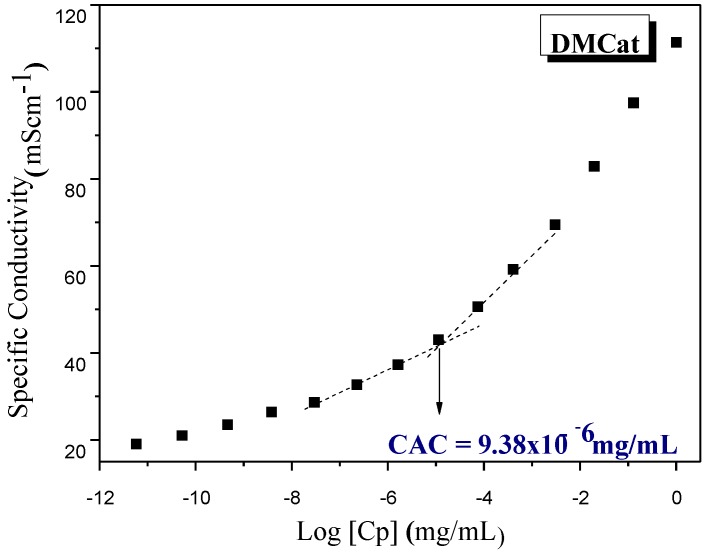
Graphic of specific conductivity versus function of logarithm concentration of DMCat of 1 mg/mL to 1 × 10^−6^ mg/mL. DMCat sample solubilized in water.

The critical aggregation concentration (CAC) was evaluated with the conductivity measurements of sample aqueous solutions of DMCh and DMCat at different concentrations, resulting in CAC values of 8.9 × 10^−3^ mg/mL [37] and 9.38 × 10^−6^ mg/mL, respectively. The CAC result of the DMCat sample was calculated from the data shown in Figure 3; the result related to the DMCh sample is presented in a previous work [37]. It is also important to note that each polymer was solubilized in a different aqueous medium, according to its solubility, because the aggregation behavior of the polymers was evaluated depending on their solubility as a function of the polymer concentration, thus enabling quantification of the dilution limit that each system presents until its aggregation occurs. When it came to evaluating the stability of micellar systems, the CAC of the polymer was extremely important, as it was possible to know the minimum concentration required of the amphiphilic polymer to obtain stable micelles [18]. Therefore, the ability of the micelles to withstand the dilution is described in terms of their thermodynamic stability and micelles with low CAC values can be maintained in a more dilute solution, and therefore, thermodynamically more stable [58]. A previous study [37] on the physical chemical properties of DMCh evidenced its lower CAC as compared to non-polymeric surfactants, showing that the former was thermodynamically more stable as the micelles are formed and kept stable in more dilute solutions [58]. In the same sense, taking into account that the CAC value of DMCat was much lower as compared to that of DMCh, the former system was able to form micelles at more dilute solutions, evidencing its higher stability as compared to DMCh [59,60]. DMCat derivative was more stable than the DMCh, a fact that can be explained by the permanent positive charges present in the DMCat, as these charges prevent agglomeration of the micelles by the effect of electrostatic repulsion [37,49].

### 3.3. Drug Encapsulation Efficiency

The encapsulation efficiency (EE) of curcumin in the micellar systems of the DMCh and DMCat derivatives was evaluated through UV spectroscopy. The quantification was performed through the indirect method, from the no-encapsulated CUR contained in the supernatant. The EE values were calculated from the equation of the line obtained from the analytical curve and the results are presented in Table 1. According to the data in Table 1, both micellar systems presented high EE values. However, DMCh micelles had higher EE values when compared to DMCat. This difference may have occurred due to the difficulty of CUR crossing the barrier of positive charges as a consequence of the electronsteric effect. In addition, it was observed that all systems remained stable, with no formation of larger particles and/or particles precipitation.

### 3.4. Particle Size and Surface Charge

**Figure 4:**
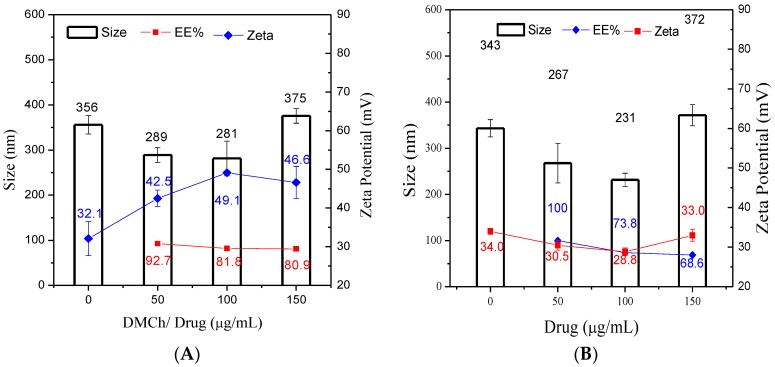
(**A**) the particle size, zeta potential, and encapsulation efficiency of micelles DMCh-CUR and (**B**) DMCat-CUR with different drug concentrations.

In case of DMCh micelles, the mean diameter of the micelles varied from 281 nm to 386 nm and from 231 to 372 nm, in case of DMCat micelles (Table 1). CUR-loaded micelles presented lower values than empty micelles (Figure 4), probably due to a contraction of the micelles in the presence of the drug, caused by the greater interaction between the hydrophobic groups pertaining to the core of the micelles and the drug [37]. However, the micelles with higher CUR encapsulation reached a limit, which in turn resulted in the expansion of the volume of the micelles to accommodate a larger amount of CUR and hydrophobic groups inside. The same behavior was observed for the micelles of DMCat, showing that the hydrophobic groups present in both micellar systems have a direct influence on the size of the micelles. Therefore, from the mean diameter results it can be stated that such micelles were promising for delivery of hydrophobic drugs via oral administration.

Zeta potential of the micelles were also evaluated and according to the data presented in Table 1, loaded and empty micelles had high positive zeta potential values (> +28 mV), regardless of whether they were made up of DMCh or DMCat. In the case of DMCh micelles, the net positive charge at the micelles surface was due to the high content of amine groups pertaining to GlcN units, which were converted to ammonium groups when the polymer was dissolved in acid medium [48]. In contrast, the surface of DMCat micelles had permanent positive charges regardless of the pH of the medium because of the presence of quaternized nitrogen atoms pertaining to the substituent groups *N*-(2-hydroxy)-propyl-3-trimethylammonium.

The zeta potential values of the empty and loaded micelles were slightly different. It was observed that the DMCh micelles showed an increase of the zeta potential with increase of the CUR loading. This behavior can be attributed to the fact that the formation of the micelles occurred by association of the hydrophobic moieties of the polymer chain to constitute the micelle core, while the hydrophilic portions of the positively charged polymer were exposed to the aqueous medium, i.e., the latter was predominantly located on the surface of the micelles. On the other hand, it was observed for DMCat micelles a small decrease on zeta potential values, which may be justified by the micelles surface, probably containing hydrophobic moieties, as well as a certain fraction of hydrophilic groups may be located in the micelles nuclei, varying the zeta potential in a narrow range. Thus, the high density of permanent positive charge in the hydrophilic shell of the micelles contributes strongly to stabilize them and also favors the interactions of the micelles with the negatively charged cell wall, resulting in the greater absorption and penetration of CUR.

According to the literature, formulations with PdI values ≤0.3 are representative of monodisperse systems [61,62]. From the PdI results of both micelles, it was possible to observe values ranging from 0.21 to 0.66 (Table 1), indicating that the systems did not present a monodisperse distribution. This fact can be justified by the non-uniform distribution of the acyl groups added to the chitosan chains, because it was a derivative from a heterogeneous reaction, which lead to the block distribution of the substituent groups. However, the values obtained were in agreement with the results obtained in the literature for chitosan derivatives [48,63,64,65].

### 3.5. Cytotoxicity Assay

**Figure 5:**
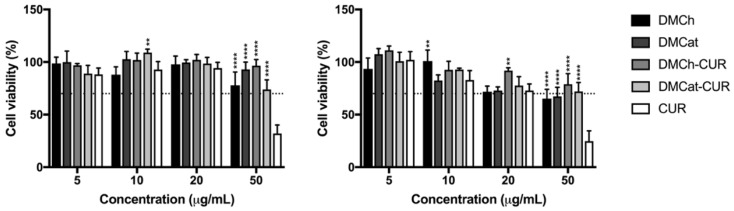
Cell viability of DMCh, DMCat, DMCh-CUR, DMCat-CUR, and free CUR against Caco-2 (**left**) and HT29-MTX (**right**) cell lines, respectively, at CUR concentrations between 5 and 50 μg/mL after 4 h of incubation. Values were reported as mean ± SD (*n* = 4). ** *p* < 0.01 and **** *p* < 0.0001 denotes a significant difference when compared with free CUR.

The in vitro cell viability studies were carried out with Caco-2 and HT29-MTX intestinal cell lines to evaluate the biocompatibility and safety for the oral administration of the empty micelles, DMCh and DMCat, the micelles loaded with CUR, DMCh-CUR and DMCat-CUR, and free CUR. Loaded micelles were incubated during 4 h with different concentrations of drug (5 to 50 μg/mL) and the correspondent amount of empty micelles was also incubated in the same conditions. The cell viability was assessed using an MTT assay (Figure 5). According to the results shown in the Figure 4, it was observed that all the tested samples had no cytotoxic effect on both cell lines for the concentrations 5, 10, and 20 μg/mL. However, at the concentration of 50 μg/mL, it was possible to note a slight decrease on cell viability for unloaded and loaded micelles. This was more evident in the case of HT29-MTX cell line, which dropped very close to 70%. This 70% of cell viability is a threshold that was considered as the cytotoxic level, according to the ISO 10993-5 guideline [52]. 

It is important to notice that CUR presented a cytotoxic effect for the highest concentration tested with statistical significance when compared with the unloaded and loaded micelles. This means that both micelles systems had the ability to maintain and protect the drug inside of the core, at least during 4 h, showing their potential to be used in the micelles for the release of the drug in the intestine, avoiding the apparent cytotoxicity of CUR. It was assumed based on our previous published studies, where similar chitosan micelles were able to retain and protect the drug, providing a hydrophobic drug release less than 20% after 4 h in the gastric and intestinal fluids [49]. The absence of cytotoxicity from the polymers was also expected taking into account our previous work [37,49]. HT29-MTX cell line showed greater sensitivity to the tested samples. However, it is known that the intestine is constituted by a small portion of these mucus-producing cells, being the majority constituted by Caco-2 cells, which presented higher cell viability levels. Taking into account these cytotoxicity results, the concentration chosen to proceed for the in vitro intestinal permeability was 20 μg/mL, since no cytotoxic effect was observed either for the free drug or for the unloaded or loaded micelles in both cell lines.

### 3.6. Permeability Studies

In order to evaluate the in vitro permeability estudies, non-toxic concentrations of free CUR and DMCh/DMCat loaded with CUR were selected based on cell viability results. Tests were carried out uni-directionally from the apical to the basolateral compartment. Therefore, a monoculture model consisting of Caco-2 cells was used, which represents the standard model that mimics human enterocytes. In addition, the triple co-culture model, Caco-2/HT29-MTX:Raji B was used due to be a model that better mimic the human intestine when compared to isolated Caco-2 cells, since HT29-MTX are secretory cells of mucus and Raji B cells can induce differentiation of the Caco-2 cell phenotype into M cells. 

**Figure 6:**
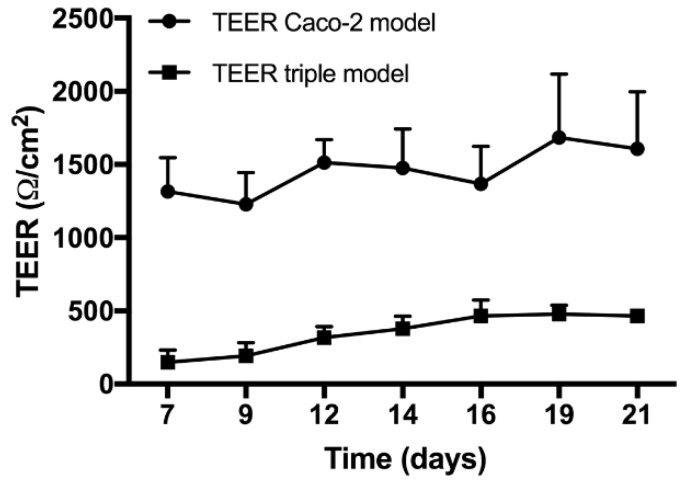
Transepithelial electrical resistance (TEER) values (Ω/cm^2^) monitored in function of time during the 21 days of culture on Transwell™ membranes for Caco-2 monoculture model and Caco-2/HT29-MTX:Raji B triple co-culture model.

The monolayer’s integrity and growth were carefully verified before the day of the experiment by measuring the TEER regularly during 21 days in order to make sure that a confluent monolayer was formed (Figure 6). As can be seen in Figure 6, Caco-2 model presented much higher TEER values compared to the triple model. These higher values were expected due to the tight junctions presented in Caco-2 cells [66]. On the other hand, the triple model presented lower TEER values due to the presence of HT29-MTX. Both models presented TEER values after 21 days that supported those monolayers to be used in the permeability experiment.

**Figure 7:**
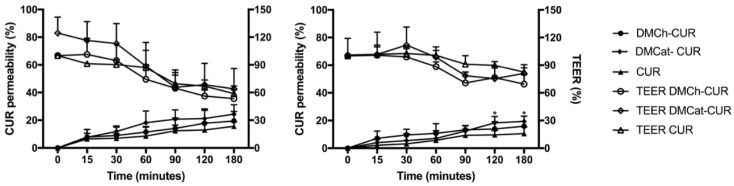
In vitro permeability profile of DMCh-CUR, DMCat-CUR, and free CUR across Caco-2 monoculture model (**left**) and across Caco-2/HT29-MTX:Raji B triple co-culture model (**right**) expressed in percentage. All experiments were conducted from the apical to basolateral compartment in 1% (*v*/*v*) DMSO in HBSS at 37 °C. Error bars represent mean ± SD (*n* = 3) and * *p* < 0.05 denotes a significant difference between DMCat-CUR and CUR.

TEER values before starting the experiment were about 1600 Ω/cm^2^ in the case of Caco-2 model and up to 450 Ω/cm^2^ in the case of the triple model (Figure 6). The TEER values were normalized in percentage by the value at the beginning of the experiment (time zero). The permeability profile of CUR permeated through both models was plotted as a function of time expressed in percentage and is shown in Figure 7. It is possible to observe the permeability of CUR across Caco-2 monoculture model ranged from around 15% in the case of free CUR to around 18% and 24% for DMCh-CUR and DMCat-CUR, respectively, showing that both polymers are capable of carrying CUR. However, the micellar system constituted by DMCat-CUR presented no statistical difference in terms of permeability through monoculture compared to DMCh-CUR, as the first one appeared to have a faster CUR release over time, reaching almost the same permeability after 60 min compared to the CUR permeability at 180 min. The triple co-culture model presented a permeability value in the range of 10% in case of free CUR to 16% and 19% in the case of DMCat-CUR and DMCh-CUR, respectively.

In this model, a more controlled permeability of CUR was observed over time possibly due to the presence of mucus, which may delay the transport of CUR through the intestinal barrier. In addition, and as can be concluded, there is no significative difference in CUR permeability between both micellar systems. However, for the triple model, DMCat-CUR presented a statistical significance compared with free CUR, evidencing that this system presents a greater capacity to permeate in a more controlled way through the biological barriers and to do the release of the drug. 

The decrease on TEER values in Caco-2 monoculture model were in agreement with the higher permeability observed in this mode. The triple co-culture model presented lower CUR permeability values for all the samples tested and this may be due to two important factors to consider due to the complexity of evaluating different biological systems. First, TEER values presented at the end of the 21 days for the triple model presented a lower value (below 500 Ω/cm^2^) than that observed in the co-culture (above 1500 Ω/cm^2^), making it impossible to compare between the two models. Second, the presence of mucus that entrapes the free CUR avoiding their passage through the monolayer. The lower ability of the micellar systems to permeate through the biological barriers is explained by the high capacity of mucoadhesion that the polymer presents. Preliminary studies show that the DMCat sample had a mucoadhesion capacity greater than the chitosan [67,68,69], due to the permanent charges present in the polymer chain (DMCat), emphasizing the importance of electrostatic forces for the establishment of interaction with the biological substrate. The effect of mucoadhesion is responsible for maintaining the DMCat-CUR system on the surface of the biological barrier, allowing the release of the sustained release of the drug over time, and in this case, the release of the drug occurs through degradation of the carrier or by diffusion through the polymeric wall. In the process of degradation are involved lysozyme and/or *N*-acetyl-beta-d-glucosaminidase (NAGase), which are enzymes present in the human body, capable of performing the hydrolysis of 1,4-beta-linkages between *N*-acetyl-d-glucosamine, a constituent of polymer chains of chitosan derivatives [67,70,71]. Perhaps, a longer experiment would be necessary to verify if CUR release from DMCat-CUR and subsequent CUR permeation would increase with time. Still, DMCat-CUR presented a more sustained and higher CUR intestinal permeability, which according to the previous results, demonstrate this system as a potential CUR intestinal delivery system.

## 4. Conclusions

Modification of the derivative 3,6-*O*,*O*′-dimyristoyl chitosan by the addition of quaternary groups was conducted to improve the solubility of the promising system to carry hydrophobic drugs; it was successfully performed. The presence of the permanent positive charges in the DMCat derivative allowed the solubilization of the polymer over a wide pH range. In addition, the characteristics of this polymer allowed the recognition thereof by the immunological system, thus facilitating its passage through the biological barriers, allowing the circulation of the polymer charged with a hydrophobic drug of interest.

## Figures and Tables

**Table 1:** Size, polydispersity index, zeta potential, and encapsulation efficiency of DMCh and DMCat micelles.

Drug (μg)	Micelles–CUR							
	Sample DMCh				Sample DMCat			
	Size (nm) ^1^	PDI ^2^	Zeta (mV) ^3^	EE (%) ^4^	Size (nm) ^1^	PDI ^2^	Zeta (mV) ^3^	EE (%) ^4^
0	356 ± 21	0.53 ± 0.03	32.1 ± 4.4	-	343 ± 19	0.21 ± 0.02	34.0 ± 0.8	-
50	289 ± 17	0.49 ± 0.04	42.5 ± 2.1	92.7 ± 0.8	268 ± 43	0.62 ± 0.01	30.5 ± 1.2	100 ± 0.5
100	281 ± 38	0.44 ± 0.04	49.1 ± 0.2	81.8 ± 1.0	231 ± 15	0.66 ± 0.02	28.8 ± 1.0	73.8 ± 1.0
150	386 ± 168	0.44 ± 0.16	46.6 ± 4.12	80.9 ± 0.9	372 ± 23	0.49 ± 0.03	33.0 ± 1.5	68.6 ± 0.9

Size ^1^, polydispersity index ^2^, and zeta potential ^3^ were determined by carrying out dynamic light scattering measurements. Encapsulation efficiency ^4^ of curcumin (CUR) in percentage. Values were reported as mean ± SD (*n* = 3).

## References

[1] Crucho C.I.C., Barros M.T. (2017). Polymeric nanoparticles: A study on the preparation variables and characterization methods. Mater. Sci. Eng. C.

[2] Lu Z.R., Qiao P. (2018). Drug Delivery in Cancer Therapy, Quo Vadis?. Mol. Pharm..

[3] Anitha A., Maya S., Deepa N., Chennazhi K.P., Nair S.V., Tamura H., Jayakumar R. (2011). Efficient water soluble O-carboxymethyl chitosan nanocarrier for the delivery of curcumin to cancer cells. Carbohydr. Polym..

[4] Sajomsang W., Gonil P., Saesoo S., Ruktanonchai U.R., Srinuanchai W., Puttipipatkhachorn S. (2014). Synthesis and anticervical cancer activity of novel pH responsive micelles for oral curcumin delivery. Int. J. Pharm..

[5] Ghalandarlaki N., Alizadeh A.M., Ashkani-Esfahani S. (2014). Nanotechnology-applied curcumin for different diseases therapy. Biomed. Res. Int..

[6] Sarisozen C., Abouzeid A.H., Torchilin V.P. (2014). The effect of co-delivery of paclitaxel and curcumin by transferrin-targeted PEG-PE-based mixed micelles on resistant ovarian cancer in 3-D spheroids and in vivo tumors. Eur. J. Pharm. Biopharm..

[7] Popat A., Karmaka S.R., Jambhrunkar S., Xu C., Yu C. (2014). Curcumin-cyclodextrin encapsulated chitosan nanoconjugates with enhanced solubility and cell cytotoxicity. Colloids Surf. B Biointerfaces.

[8] Aggarwal B.B., Gupta S.C., Sung B. (2013). Curcumin: An orally bioavailable blocker of TNF and other pro-inflammatory biomarkers. Br. J. Pharmacol..

[9] Khan S., Imran M., Butt T.T., Ali Shah S.W., Sohail M., Malik A., Das S., Thu H.E., Adam A., Hussain Z. (2018). Curcumin based nanomedicines as efficient Nanoplatform for treatment of cancer: New developments in reversing cancer drug resistance, rapid internalization, and improved anticancer efficacy. Trends Food Sci. Technol..

[10] Rauf A., Imran M., Orhan I.E., Bawazeer S. (2018). Health perspectives of a bioactive compound curcumin: A review. Trends Food Sci. Technol..

[11] Nelson K.M., Dahlin J.L., Bisson J., Graham J., Pauli G.F., Walters M.A. (2017). The Essential Medicinal Chemistry of Curcumin. J. Med. Chem..

[12] Anand P., Kunnumakkara A.B., Newman R.A., Aggarwal B.B. (2007). Bioavailability of Curcumin: Problems and Promises. Mol. Pharm..

[13] Angeles L., Angeles L., Haven N. (2016). Phase 1 study of PSMA-targeted docetaxel-containing nanoparticle BIND-014 in patients with advanced solid tumors. Clin. Cancer Res..

[14] Kyung H., Minkyu A., Sun J., Sym J. (2014). A phase II trial of Cremorphor EL-free paclitaxel (Genexol-PM) and gemcitabine in patients with advanced non-small cell lung cancer. Cancer Chemother. Pharmacol..

[15] Cortez A.J., Tudre P.J., Kujawa K.A., Lisowska K.M. (2018). Advances in ovarian cancer therapy. Cancer Chemother. Pharmacol..

[16] Tien C.L., Lacroix M., Ispas-Szabo P., Mateescu M.A. (2003). *N*-acylated chitosan: Hydrophobic matrices for controlled drug release. J. Control. Release.

[17] Ishihar M.A., Kishimoto M., Fujita S., Hattori H., Kanatani Y. (2012). Biological, Chemical and Physical Compatibility of Chitosan and Biopharmaceuticals. Chitosan-Based Syst. Biopharm. Deliv. Target. Polym. Ther..

[18] Huo M., Zhang Y., Zhou J., Zou A., Li J. (2011). Formation, microstructure, biodistribution and absence of toxicity of polymeric micelles formed by *N*-octyl-*N*,*O*-carboxymethyl chitosan. Carbohydr. Polym..

[19] Matsumura Y., Kataoka K. (2009). Preclinical and clinical studies of anticancer agent-incorporating polymer micelles. Cancer Sci..

[20] Nishiyama N., Kataoka K. (2006). Current state, achievements, and future prospects of polymeric micelles as nanocarriers for drug and gene delivery. Pharmacol. Ther..

[21] Gao J., Ming J., He B., Fan Y., Gu Z., Zhang X. (2008). Preparation and characterization of novel polymeric micelles for 9-nitro-20(*S*)-camptothecin delivery. Eur. J. Pharm. Sci..

[22] Muley P., Kumar S., El Kourati F., Kesharwani S.S., Tummala H. (2016). Hydrophobically modified inulin as an amphiphilic carbohydrate polymer for micellar delivery of paclitaxel for intravenous route. Int. J. Pharm..

[23] Ito T., Yoshida C., Murakami Y. (2013). Design of novel sheet-shaped chitosan hydrogel for wound healing: A hybrid biomaterial consisting of both PEG-grafted chitosan and crosslinkable polymeric micelles acting as drug containers. Mater. Sci. Eng. C.

[24] Du Y., Cai L., Liu P., You J., Yuan H., Hu F. (2012). Biomaterials Tumor cells-speci fi c targeting delivery achieved by A54 peptide functionalized polymeric micelles. Biomaterials.

[25] Jiao J., Li X., Zhang S., Liu J., Di D., Zhang Y., Zhao Q., Wang S. (2016). Redox and pH dual-responsive PEG and chitosan-conjugated hollow mesoporous silica for controlled drug release. Mater. Sci. Eng. C.

[26] Elsaid Z., Taylor K.M.G., Puri S., Cath A., Al-jamal K., Bai J., Klippstein R., Wang T., Forbes B., Chana J. (2017). Mixed micelles of lipoic acid-chitosan-poly(ethylene glycol) and distearoylphosphatidylethanolamine-poly(ethylene glycol) for tumor delivery. Eur. J. Pharm. Sci..

[27] Jin X., Li N., Ju C., Sun M., Zhang C., Pin Q.G. (2011). Biomaterials The mechanism of enhancement on oral absorption of paclitaxel by *N*-octyl-*O*-sulfate chitosan micelles. Biomaterials.

[28] Huo M., Liu Y., Wang L., Yin T., Qin C., Xiao Y., Yin L., Liu J., Zhou J. (2016). Redox-sensitive micelles based on *O*,*N*-hydroxyethyl chitosan-octylamine conjugates for triggered intracellular delivery of paclitaxel Redox-sensitive micelles based on *O*,*N*-hydroxyethyl chitosan-octylamine conjugates for triggered intracellular deliver. Mol. Pharm..

[29] Attia E.A.B., Ong Z.Y., Hedrick J.L., Lee P.P., Lee P.L.R., Hammond P.T., Yang Y.-Y. (2011). Mixed micelles self-assembled from block copolymers for drug delivery. Curr. Opin. Colloid Interface Sci..

[30] Jiang G.B.B., Quan D., Liao K., Wang H. (2006). Novel polymer micelles prepared from chitosan grafted hydrophobic palmitoyl groups for drug delivery. Mol. Pharm..

[31] Zhang C., Qu G., Sun Y., Wu X., Yao Z., Guo Q., Ding Q., Yuan S., Shen Z., Ping Q. (2008). Pharmacokinetics, biodistribution, efficacy and safety of *N*-octyl-*O*-sulfate chitosan micelles loaded with paclitaxel. Biomaterials.

[32] Hu F.Q.Q., Zhao M.D.D., Yuan H., You J., Du Y.Z.Z., Zeng S. (2006). A novel chitosan oligosaccharide-stearic acid micelles for gene delivery: Properties and in vitro transfection studies. Int. J. Pharm..

[33] Chenguang L.I.U., Desai K.G., Xiguang C., Hyun-jin P. (2005). Preparation and Charaterization of Self-assembled Nanoparticles Based on Linolenic-acid Modified Chitosan. J. Ocean Univ. China.

[34] Hu F.Q., Ren G.F., Yuan H., Du Y.Z., Zeng S. (2006). Shell cross-linked stearic acid grafted chitosan oligosaccharide self-aggregated micelles for controlled release of paclitaxel. Colloids Surf. B Biointerfaces.

[35] Huo M., Zhang Y., Zhou J., Zou A., Yu D., Wu Y., Li J., Li H. (2010). Synthesis and characterization of low-toxic amphiphilic chitosan derivatives and their application as micelle carrier for antitumor drug. Int. J. Pharm..

[36] Du Y.Z.Z., Wang L., Yuan H., Wei X.H.H., Hu F.Q.Q. (2009). Preparation and characteristics of linoleic acid-grafted chitosan oligosaccharide micelles as a carrier for doxorubicin. Colloids Surf. B Biointerfaces.

[37] Silva D.S., Almeida A., Prezotti F., Cury B., Campana-Filho S.P., Sarmento B. (2017). Synthesis and characterization of 3,6-*O*,*O*’-dimyristoyl chitosan micelles for oral delivery of paclitaxel. Colloids Surf. B Biointerfaces.

[38] Pan Z., Gao Y., Heng L., Liu Y., Yao G., Wang Y., Liu Y. (2013). Amphiphilic *N*-(2,3-dihydroxypropyl)-chitosan-cholic acid micelles for paclitaxel delivery. Carbohydr. Polym..

[39] Di Martino A., Sedlarik V. (2014). Amphiphilic chitosan-grafted-functionalized polylactic acid based nanoparticles as a delivery system for doxorubicin and temozolomide co-therapy. Int. J. Pharm..

[40] Zhang C., Ding Y., Lucy L., Ping Q. (2007). Polymeric micelle systems of hydroxycamptothecin based on amphiphilic *N*-alkyl-*N*-trimethyl chitosan derivatives. Colloids Surf. B Biointerfaces.

[41] Zuñiga A., Debbaudt A., Albertengo L., Rodríguez M.S. (2010). Synthesis and characterization of *N*-propyl-*N*-methylene phosphonic chitosan derivative. Carbohydr. Polym..

[42] Woraphatphadung T., Sajomsang W., Gonil P., Treetong A., Akkaramongkolporn P., Ngawhirunpat T., Opanasopit P. (2016). pH-Responsive polymeric micelles based on amphiphilic chitosan derivatives: Effect of hydrophobic cores on oral meloxicam delivery. Int. J. Pharm..

[43] Wu J., Wei W., Wang L.Y., Su Z.G., Ma G.H. (2007). A thermosensitive hydrogel based on quaternized chitosan and poly(ethylene glycol) for nasal drug delivery system. Biomaterials.

[44] Sonia T.A., Sharma C.P. (2011). In vitro evaluation of *N*-(2-hydroxy) propyl-3-trimethyl ammonium chitosan for oral insulin delivery. Carbohydr. Polym..

[45] Ruihua H., Bingchao Y., Zheng D., Wang B. (2012). Preparation and characterization of a quaternized chitosan. J. Mater. Sci..

[46] Santos D.M., Bukzem A.L., Campana-Filho S.P. (2016). Response surface methodology applied to the study of the microwave-assisted synthesis of quaternized chitosan. Carbohydr. Polym..

[47] Rinaudo M., Milas M., Le Dung P. (1993). Characterization of chitosan. Influence of ionic strength and degree of acetylation on chain expansion. Int. J. Biol. Macromol..

[48] Almeida A., Silva D.S., Gonçalves V., Sarmento B. (2017). Synthesis and characterization of chitosan-grafted-polycaprolactone micelles for modulate intestinal paclitaxel delivery. Drug Deliv. Transl. Res..

[49] Silva D.S., Almeida A., Prezotti F.G., Facchinatto W.M., Colnago L.A., Campana-Filho S.P., Sarmento B. (2017). Self-aggregates of 3,6-*O*,*O*’-dimyristoylchitosan derivative are effective in enhancing the solubility and intestinal permeability of camptothecin. Carbohydr. Polym..

[50] Hirai A., Odani H., Nakajim A.A. (1991). Determination of degree of deacetylation of chitosan by ^1^H NMR spectroscopy. Polym. Bull..

[51] Fiamingo A., Delezuk J.A.D.M., Trombotto S., David L., Campana-Filho S.P. (2016). Extensively deacetylated high molecular weight chitosan from the multistep ultrasound-assisted deacetylation of beta-chitin. Ultrason. Sonochem..

[52] (2009). ISO/EN10993-5. International Standard ISO 10993-5 Biological Evaluation of Medical Devices—Part 5: Tests for Cytotoxicity: In Vitro Methods.

[53] Araújo F., Sarmento B. (2013). Towards the characterization of an in vitro triple co-culture intestine cell model for permeability studies. Int. J. Pharm..

[54] Antunes F., Andrade F., Araújo F., Ferreira D., Sarmento B. (2013). Establishment of a triple co-culture in vitro cell models to study intestinal absorption of peptide drugs. Eur. J. Pharm. Biopharm..

[55] Brugnerotto J., Lizardi J., Goycoolea F.M., Argüelles-Monal W., Desbrières J., Rinaudo M. (2001). An infrared investigation in relation with chitin and chitosan characterization. Polymer.

[56] Cho J., Grant J., Piquette-miller M., Allen C. (2006). Synthesis and Physicochemical and Dynamic Mechanical Properties of a Water-Soluble Chitosan Derivative as a Biomaterial. Biomacromolecules.

[57] Xiao B., Wan Y., Wang X., Zha Q., Liu H., Qiu Z., Zhang S. (2012). Synthesis and characterization of *N*-(2-hydroxy)propyl-3-trimethyl ammonium chitosan chloride for potential application in gene delivery. Colloids Surf. B Biointerfaces.

[58] Croy S.R., Kwon G.S. (2006). Polymeric micelles for drug delivery. Curr. Pharm. Des..

[59] Li Y., Li L., Dong H., Cai X., Ren T. (2013). Pluronic F127 nanomicelles engineered with nuclear localized functionality for targeted drug delivery. Mater. Sci. Eng. C.

[60] Li X., Yu Y., Ji Q., Qiu L. (2014). Targeted delivery of anticancer drugs by aptamer AS1411 mediated Pluronic F127/cyclodextrin-linked polymer composite micelles. Nanomedicine.

[61] Rao J.P., Geckeler K.E. (2011). Polymer nanoparticles: Preparation techniques and size-control parameters. Prog. Polym. Sci..

[62] Hickey J.W., Santos J.L., Williford J.M., Mao H.Q. (2015). Control of polymeric nanoparticle size to improve therapeutic delivery. J. Control. Release.

[63] Yuan H., Lu L., Du Y., Hu F. (2010). Stearic acid-g-chitosan polymeric micelle for oral drug delivery: In intro transport and in vivo absorption. Mol. Pharm..

[64] Zhang C., Ping Q., Zhang H., Shen J. (2003). Preparation of *N*-alkyl-*O*-sulfate chitosan derivatives and micellar solubilization of taxol. Carbohydr. Polym..

[65] Zhang C., Qineng P., Zhang H. (2004). Self-assembly and characterization of paclitaxel-loaded *N*-octyl-*O*-sulfate chitosan micellar system. Colloids Surf. B Biointerfaces.

[66] Lechanteur A., Almeida A., Sarmento B. (2017). Elucidation of the impact of cell culture conditions of Caco-2 cell monolayer on barrier integrity and intestinal permeability. Eur. J. Pharm. Biopharm..

[67] Park J.H., Saravanakumar G., Kim K., Kwon I.C. (2010). Targeted delivery of low molecular drugs using chitosan and its derivatives. Adv. Drug Deliv. Rev..

[68] Hejazi R., Amiji M. (2003). Chitosan-based gastrointestinal delivery systems. J. Control. Release.

[69] Tozaki H., Komoike J., Tada C., Maruyama T., Terabe A., Suzuki T., Yamamoto A., Muranishi S. (1997). Chitosan capsules for colon-specific drug delivery: Improvement of insulin absorption from the rat colon. J. Pharm. Sci..

[70] Bernkop-Schnürch A., Dünnhaupt S. (2012). Chitosan-based drug delivery systems. Eur. J. Pharm. Biopharm..

[71] Chauvierre C., Leclerc L., Labarre D., Appel M., Marden M.C., Couvreur P., Vauthier C. (2007). Enhancing the tolerance of poly(isobutylcyanoacrylate) nanoparticles with a modular surface design. Int. J. Pharm..

